# Just Like Any Other Family? Everyday Life Experiences of Mothers of Adults with Severe Mental Illness in Sweden

**DOI:** 10.1007/s10597-020-00549-z

**Published:** 2020-01-08

**Authors:** Katarina Piuva, Helene Brodin

**Affiliations:** Department of Social Work, Stockholm University, 106 91 Stockholm, Sweden

**Keywords:** Caring experience, Experiential knowledge, Mothers, Adult children, Severe mental illness, Sanism

## Abstract

This study explores experiences of mothers in Sweden who care for their adult children suffering from severe mental illness. Using 15 interviews with mothers from 40 to 80 years old, the article examines how predominant professional knowledge and sanism constructs the mothers and their children as deviant and what counterstrategies the mothers develop as a response to these experiences of discrimination. The findings show that the mothers’ experiences are characterized by endless confrontations with negative attitudes and comments that have forced them to go through painful and prolonged processes of self-accusations for not having given enough love, care, support and help in different stages of their children's life. But the mothers’ experiences also reveal important aspects of changes over the life span. As the mothers are ageing, the relationship between them and their children becomes more reciprocal and the ill child may even take the role as family carer.

## Introduction

As in the majority of advanced welfare states, the mental health care system in Sweden has shifted from in-hospital psychiatric care to community care with support from professional caregivers. Official statistics reflect this change in mental health care. From the end of the 1960s until the middle of the 1980s, in-hospital psychiatric beds in Sweden declined by 80% (SOU 1992), and from 1999 to 2009, in-hospital beds decreased by an additional 23% (National Board of Health and Welfare 2010). Politically, this huge transformation of the mental health care system has been motivated by an effort to support the independence and inclusion of mental health service users in society (Government Bill 1993/94). However, studies also show that the decrease of hospitalised care has a downside: people who suffer from mental illnesses are often left to cope on their own without adequate professional help and support (Lindgren et al. 2014). When the professional system fails to meet peoples’ needs, the responsibility to provide care falls on family members. This is especially true for mothers. International, as well as Swedish studies, indicate that mothers usually take on a life-long and round-the-clock caring responsibility for children with severe mental illness (SMI), even though their child may be an adult when he or she becomes ill (Austin and Carpenter, 2008; Johansson e t al. 2010; Leiter et al. 2004; Milliken 2001). Regardless of the age of the child, mothers of children with mental illnesses usually cut down their working hours or even quit their employment to take care of the ill child. Mothers are also more involved in personal care than fathers, who instead are more engaged in financial support and medical treatments (Gosh and Greenberg 2012).

Though research shows that close relationships and practical and emotional support from family members are vital for the recovery process (Reupert et al. 2015), studies indicate that many parents feel that professionals blame them for causing the illness and that their caring responsibilities and support are neither recognised nor wanted by providers of health and social care services (Gosh and Greenberg 2012; Johansson et al. 2010; Milliken and Nortcott 2003; Nyström and Svensson 2004). In addition, in Sweden, which has had comparatively abundant health and social care services, both policy and research have ignored the caring experiences of mothers and fathers of adult children (Grassman et al. 2009).

Against this backdrop, this article aims to explore the experiences of mothers in Sweden who care for adult children suffering from SMI. We focus on the mothers, as previous studies show that mothers usually take the largest caring responsibility. Three questions have guided this study: how has the experience of having a child with SMI affected family relations? How has the child’s condition affected employment and family income? How have the mothers experienced interactions with the mental health care system?

The article starts with a brief overview of how mental illness may affect and transform parenthood and family life. The study then continues with a presentation of methods and data followed by findings from the interview data. In the concluding part of the article, we contextualise the findings with respect to the development of mental health services and what professionals can learn from the experiences of mothers.

## Sanism and the Ignorance of Experiential Knowledge of Severe Mental Illness

Many studies have described how SMI affects family life and transforms the parental role (Austin and Carpenter 2008; Johansson et al. 2010; Milliken 2001; Milliken and Nortcott 2003; Nyström and Svensson 2004; Sanders et al. 2014; Veltman et al. 2002). In most cases, the transformed parental role is negatively conceptualised and described as a lifelong burden characterised by shame, guilt and humiliation (Grey et al. 2010; Larsen and Corrigan 2008; Van der Sanden et al. 2013). Traditionally, in western societies, professional knowledge has had a higher status than knowledge gained through the lived experience of mental illness and psychiatric care (Faulkner 2017). Throughout the twentieth century, mainstream research on mental illness and its consequences for family life emphasised the increased care burden following mental illness (Gubman and Tessler 1987), and mental illness was generally viewed as a phenomenon that ended in dysfunctional family relations. Moreover, the cause of the mental illness was often described as rooted in family life in general and in the relationship between the mother and the child in particular. However, recent developments in psychiatric science have removed the blame of the family by focussing on biochemistry and genetics. Although the biomedical model still subordinates parents’ experiences to professional knowledge, it has contributed to radically question and change the pathological perception of family relations and mothers of children with SMI. Furthermore, contemporary research shows that most families endure whatever tensions that might follow from a child suffering from SMI and that most families successfully adapt to the situation (Schön et al. 2009; Topor 2006). Some studies even indicate that for some parents, SMI can develop into a positive experience including new meanings and values in life (Veltman et al. 2002). In addition, research indicates that parents and other family members play an essential role in the process of recovering from SMI (Reupert et al. 2015).

Regardless of whether the field of research about family experiences of SMI and psychiatric care emphasises burden and distress, adaption to the situation or positive experiences of recovery, family members still fight against the dominant power of professional knowledge. The labelling and stigmatisation of mental illness originate from the discursive power of medical science (Perlin and Dorfman 1993). Michael Foucault has expressed the essential meaning of psychiatric discursive power, saying ‘the language of psychiatry is a monologue of reason about madness’ (Foucault 1965). This discursive power has also resulted in oppressive practices and ignorance of survival knowledge (Le Francois et al. 2013). Drawing on the works of Chamberlin (e.g., 1979), Poole et al. (2012) define this discursive power as ‘sanism’. Sanism includes expectations and professional judgements that people with mental illness are incompetent, constantly in need of supervision and assistance, unpredictable, violent and irrational. In addition, sanism is seldom overt but commonly suffered as multiple, everyday experiences of insults, injustices and indignities (Poole et al. 2012) and/or manifested as fear, social distancing, disgust and avoidance (Overton and Medina 2008). These practices of sanism also spill over on family members of people with SMI (Van der Sanden et al. 2013).

In this article, we explore how discourses and practices of sanism, such as the priority of professional knowledge, the subordination of survival perspectives, prejudice and social distancing shape the everyday experiences of mothers of adult children suffering from SMI. Our approach is to develop an understanding of how sanism constructs the mothers and their children as being ‘the deviant family’. In addition, we also examine what strategies the mothers have developed to deal with sanism in Swedish society.

## Methods

The empirical basis of this paper consists of 15 in-depth interviews with mothers to adult children with SMI. All children had been diagnosed with a mental illness when they were adolescents or young adults (16 to 22 years old). At the time of the interviews, the mothers aged from 40 to 80 years old and their children aged from 20 to 50 years old. Thus, the older mothers and their children had had their first encounters with mental healthcare services in the 1960s and the 1970s, while the younger mothers’ experiences were from recent years. The interviews with the mothers thus reflect both past and present experiences of interactions with the mental healthcare system. Moreover, they mirror past and present encounters with negative attitudes and prejudices towards mental illness in Sweden. Most of the mothers were married and shared the responsibilities for the children with their fathers. A few of the mothers were divorced and were therefore almost exclusively alone with the responsibilities for their children’s well-being.

Contact with the mothers was accessed through two user organisations situated in Stockholm, the National Association for Social and Mental Health (*Riksförbundet för Social och Mental Hälsa*, RSMH) and the Association for Schizophrenia (*Schizofreniförbundet*). This strategy may have affected our findings. All participants were members of the user organisations and it is therefore reasonable to assume that the mothers of our study had more elaborated strategies to deal with sanism than parents who are non-members.

All interviews were performed in accordance with a semi-structured topic guide based on a series of guiding themes that we wanted the mothers to describe and identify. This use of an open-ended semi-structured topic guide enabled a ‘natural conversation’ guided by the research questions, a method that generated rich and thick descriptions of the lived experiences and perceptions of the mothers (cf. Gillham 2000). The topic guide included the following themes: (1) the diagnosis of the child and how it has changed over time; (2) positive and negative experiences of care and support; (3) family network; (4) significance of the illness for choices in life, career and relationships; (5) crises in private life and in working life and (6) perspectives on the future.

The interviews were transcribed *verbatim* and analysed in accordance with a narrative approach. As the narrative is a significant means for individuals to express and give meaning to personal experiences, narrative analysis has proven to be a useful methodological approach in studies of life experiences (Bamberg and Andrews 2004). On the basis of the themes in the topic guide, the mothers’ narratives of experiences of caring for their children were analysed and compared from the perspective of storylines (experiences of exclusion and inclusion), language use and critical incidents (experiences and encounters with attitudes and prejudices against mental illness in various arenas and settings) and relation to the context (welfare reforms and disability policies). The analysis of the interview data is summarised in the following themes: the turning point, experiences of social exclusion, experiences of marginalisation at work, provision of financial help and support, encounters with in-hospital psychiatric care, encounters with community care and looking ahead.

## Results

### The Turning Point

Mourning, loss and the sense of a never-ending burden of care were central themes in the mothers’ descriptions of their early experiences of their children’s mental illnesses. The first time the mothers began to realise that there was something different with their children was therefore defined as a period of great anxiety and worry. The mothers also described how the whole family was negatively affected. The siblings of the ill children were neglected and, in many cases, conflicts between the parents emerged. Nearly all mothers mentioned how they began to fear the future. The future suddenly appeared as beyond control, unpredictable and dangerous. Accusations and searching for an answer to the illness was one recurrent theme in all the mothers’ stories how they experienced the first years of the child’s illness. One mother summarised her thoughts during the first years as:

When you’ve a child with this disease, then of course you wonder where he got it from? How could it be this way? And then, my first thought was to look at myself and consider if I have done anything wrong? Didn’t he get enough love? Perhaps I didn’t see his problems when he was a child. Did I miss something? Didn’t I do enough for him?

Other frequent themes in the mothers’ descriptions of the first years when the child became ill, was the fact that neither the child nor the family was like other families. However, as our interviews included the mothers’ experiences from the point when the children became ill to the present situation, another and an alternative perspective also emerged in the interviews. This alternative perspective included change of values, perceptions and new approaches towards life. Therefore, most of our interviews were not stories of never-ending grief. Instead, one central theme in the mothers’ experiences was how an unexpected incident in life resulted in re-evaluations of friendship, family life and life goals. Here, we traced two different routes in the mothers’ life stories that described different ways out of the turning point in life triggered by the child’s mental illness. One way out was separation and the breaking-up of the original family. The breaking-up of the original family both included separation from the father of the child and from grandparents or one or several of the siblings. The other way out in the mother’s stories was closer bonding in the original family. In the mothers’ life stories characterised by closer bonding, the children played a central role – but not only from a one-sided caregiving relationship. On the contrary, sometimes the mothers described how the relationship with the child had developed into a friendship based on unique experiences and reciprocal relationships of giving and taking.

Regardless of whether the child’s illness had resulted in separation or closer bonding of the family, the main thread in the mothers’ stories was that their experiences had helped them to distinguish between small and big issues in life. One of the mothers emphasised that the illness could actually be understood as a gift that had taught her to value the right things in life. Another mother concluded this experience as: ‘You don’t exactly go ballistic because you lack table linen in matching colours’.

## Experiences of Social Exclusion

Another frequent theme in the interviews with the mothers was that people had responded with negative reactions and were withdrawn when they mentioned that their child was suffering from a mental illness. For example, instead of being supportive and offering help, friends and acquaintances stopped keeping in touch or even disappeared when the child became ill. Pretending that the ill child did not exist was another reaction the mothers described. One mother, who had a son who had suffered from schizophrenia for more than 20 years, mentioned how much it still hurt when friends and acquaintances pretended her son no longer existed. She described how people often asked her about her other children and how they were doing, but never with a single word mentioned her son, who had schizophrenia.

Some people they run away, you can say, when something as this happens. They have never asked about our son. And that feels very hard. They may ask how things are with our other son, and how many children he has, and our daughter, and how things are going for her, but our son, who is ill, he’s never mentioned, he doesn’t exist.

Another mother provided us with the expression ‘social degradation’ in her description of how her surroundings had reacted to her child’s illness. Her strategy to deal with these negative encounters was to redefine her entire network. Therefore, she experienced the degradation as temporary and chose to interpret this experience as some people in her social network not sharing her values of what was important in life. After this experience, she became much more careful in choosing friends.

Other negative reactions described by the mothers were the neighbours’ behaviours. One recurrent theme was that the mothers felt uncomfortable with neighbours and the area where they lived, especially if they lived in the same neighbourhood as they did before the child became ill. For example, one mother mentioned that when her son was diagnosed with schizophrenia, the neighbours had a garden party and one of the neighbours at that party described people suffering from SMI as ‘lunatics’ who ‘trespass in my private life’. Another mother who was more than 80 years old described how the neighbours blamed her son’s schizophrenia on her and her husband and their lack of morals and lack of upbringing their children. Though her son has lived with his illness for more than 30 years, she still notices how neighbours peek from behind the curtains when her son comes to visit and make remarks the next time they see her:

Anyway, they [the neighbours] were convinced that it was our fault. So, that’s why you had to go and feel terribly ashamed. And then—we have the bus stop just outside [our house]—“oh, I saw your son at the bus stop outside. How is he? Well, it’s such a shame”, and then they look at me…

Not only friends, acquaintances and neighbours, but also relatives have responded with negative reactions to their children’s illnesses. One of the mothers described how even her other children turned against her when her child was diagnosed with schizophrenia and accused her of being a ‘bad mother’ if she laughed or was happy. In their opinion, she had nothing more in life to be happy about.

In their stories, the mothers also returned to the fact that relatives had withdrawn and avoided contact with the child. Despite the fact that their children were adults and for many years had lived on their own, greetings and invitations to their children were addressed to them and sent to their house. The mothers pointed out that the family networks around their children had diminished considerably since they had become ill, which generated conflicts between the mothers and other relatives. In the mothers’ opinions, relatives should be able to keep in touch with their children and function as social support. However, as things had turned out, the parents were usually the only social networks their children had. One mother mentioned that she had been in a quarrel with her sister for many years because neither her sister nor her children phoned or visited her daughter who had schizophrenia, though the sister and her children lived nearby.

But it has become so delicate, because I thought and I let them know that our daughter needed them more during those first years. And my sister was very offended (…) so it became very awkward between my sister and me.

Though not explicitly mentioned by all, the mothers described in different ways how negative attitudes have made them socially isolated and marginalised. The isolation was in some cases related to self-imposed feelings of shame for their children’s behaviours. For example, one mother argued that if her daughter had behaved a little bit more ‘normal’ during her adolescence, maybe the mother and her husband would have invited people for dinner, but as the daughter usually behaved in a strange way, they withdrew from social interactions. This mother’s experience was therefore that her daughter’s mental illness hade made her alone and isolated. Another mother described the social consequences in terms of being an outcast. In this mother’s experience, the manifestation of sanism as fear of mental illness had not only affected her son but also herself as a mother.

So I really feel that way that sometimes you are an outcast. Outcast, really, literally being an outcast. I get the same feeling when I walk the streets around here that I feel (…) I am afraid that they [the neighbours] notice when our son is coming. That they wonder how he is and what’s wrong with him, really, because some people feel disgust about these things.

As nearly all mothers had experienced isolation and social exclusion by relatives, friends and neighbours, they joined a user organisation. The mothers described how membership in a user organisation had become an important strategy to deal with negative attitudes and prejudices against mental illness. The user organisations had served as a respite and a place where they had found new friends. Thus, the user organisations had enabled the mothers to build new networks that contributed to their personal well-being. However, one of the mothers said that she did not know if it was all that healthy to only spend time with people with difficult experiences. In her opinion, engagement in the user organisation had contributed to keeping the pain alive.

## Experiences of Marginalisation at Work

Even though the children became ill during late adolescence or early adulthood, most of the mothers interviewed had at some point in life chosen to work part-time to be able to carry on. In addition, many of the mothers had been unable to work for a long period. Regardless of whether they worked, were on sick leave or were retired at the point of the interview, all of the mothers described a feeling of chronic tiredness after years of fighting for adequate care for their children. Self-accusations for studying or working too much when the child was an infant or had had a difficult period during adolescence were also recurrent in the mothers’ stories. One of the mothers described how these self-accusations made her question all her ‘doings’ in life, which for many years made her degrade herself and her capacity to work. Another mother mentioned that lack of support from employers and negative attitudes from colleagues meant that her job just ‘fizzled out’ and because of this, she became unemployed.

However, support from employers appeared to have changed for the better in recent years. The oldest mothers described how they struggled with difficulties and lack of support from employers when they had to be absent from work because of the child’s illness. They also described feelings of being differently treated and how their child’s illness was met by a silence that spread at work. The silence might have been a way to show consideration, but the mothers experienced this as marginalisation and exclusion. The younger mothers described experiences that were more positive, and that employers had been supportive and forthcoming in relation to both work assignments and working hours. One of the younger mothers, however, described how she was replaced and given new work assignments when the symptoms of her daughter’s illness had escalated. She experienced this replacement as being subjected to unfair treatment:

Well, it has affected my work, you can say, so I got adjusted work assignments (…) I had to leave when our daughter became ill and we had a rather mean boss (…) It was not directly bad assignments, but I felt offended by that, really. I was offended.

The most negative experiences in relation to work were, however, about negative attitudes and reactions from colleagues. Recurrent in the mothers’ stories were experiences of not being able to talk about their families or children because of fear of how the colleagues would respond. For example, one mother described how she never dared to tell anyone at work about her child who had schizophrenia. At the time when her child became ill, there was much media coverage about the supposed violent behaviour of people with schizophrenia. Several of her colleagues had responded by making negative remarks and comments about mental illness. Therefore, she never spoke of her family or her children at work. In the end, her colleagues’ negative attitudes and prejudices made her feel very lonely and isolated. Not until she retired did she dare to tell a few of her colleagues of her child’s illness:

And you know I could never talk about my children at work. Never! Then when he [her son] finally received medical care and medication and all that—I could never mention that to anyone. Because what they expressed about those people in the newspaper—it revealed how they looked upon them. Therefore, you could never speak of [it]. I haven’t told anyone until now, afterwards, to a few.

To deal with colleagues’ negative attitudes and prejudices, the mothers seemed to have developed a strategy to tune in to those who had experiences similar to theirs and to confide in these persons. Therefore, if the mothers dared to talk about their families and children at work, this had usually been with colleagues they knew had similar experiences. For example, one mother mentioned how she started to cry in front of one colleague when her son had become ill and how this person supported her and took care of her. She described this person as ‘very good’ and understanding because she herself had a son who had suffered from severe depression.

## Financial Help and Support

Official reports in Sweden repeatedly point to the fact that long-term mental illness generates poverty (SOU, 2006). SMI is also one of the most common causes of disability pension in Sweden and many people suffering from SMI are entitled only to the lowest levels of benefits from Swedish social insurance. The mothers we interviewed confirmed this situation. The mothers had often provided their adult children with money. In addition, the mothers had helped to sort out the jungle of bureaucratic routines, certificates and paperwork requested to gain the benefits the child was entitled to. The mothers also described how their children had lived for years on social welfare because they had been unable to approach the psychiatrists and clinics they needed to receive benefits from the National Insurance Agency. Sometimes, the mothers violated rules and regulations just to ensure that their children received the benefits they were entitled to:

In the end I managed to get in contact with one authority official who took care of all that, though it was totally against the rules (…) that I as a mother phoned and reported the personal code number (…) took the mail in his flat and gave it to the authority official.

As their children in general lived more or less in poverty, the mothers described how they supported their children financially, even though the children in some cases were in their 50 s. The mothers who were single also described how their own financial situations were negatively affected because of this. Nevertheless, as one mother said, without her and her husband’s financial support, their child might have ended up homeless, living on the streets.

Without us I don’t understand how he would have made it (…) maybe homeless. I do not even want to think about that. It’s not only about the money; you must be able to handle them as well.

The oldest mothers who were retired emphasised that the savings they had planned to use to secure their own ageing instead were spent on their children. Some of the older mothers not only supported their children but also their grandchildren. As their son or daughter totally lacked financial resources, the mothers were forced to support their grandchildren. One of the older mothers explicitly said that if she and her husband had not had a decent income, her child’s illness would have been much more difficult to get through. This mother also reported that in order to help their son, who lived on a disability pension, her husband still worked full-time though he was more than 70 years old.

## Encounters with in-Hospital Psychiatric Care

The mothers’ experiences of encounters with psychiatric care were mixed and often depended on the year in which the child became ill. The older mothers and their children, who had their first encounters with psychiatric care in the 1960s and the 1970s, described traumatic and shocking meetings with hospital environments:

I had to leave him there. He was only 18 years, among all these (…) mostly old (…) and all the shouting (…) I saw he was afraid but I couldn’t do anything else.

The younger mothers who had children who had become ill in recent years also described the hospital environment as discouraging and almost hostile. One mother, whose daughter ended up in a psychiatric clinic after an attempted suicide, described the transfer of her daughter from somatic care to psychiatric care as a journey from paradise to hell:

It was a disaster, really, because for her it was as going from paradise to hell. Because first when she arrived to the psychiatric clinic (…) the staff at the clinic kept such a distance to the patients. They preferred to talk to the patients on three meters’ distance. They were impersonal, cold.

The same mother also mentioned that when her daughter, who at that point was in her 20 s, was admitted to the psychiatric clinic, she and her husband accompanied the daughter. The psychiatrists immediately questioned this and after three days, they were no longer allowed to speak to the doctors about their daughter. The psychiatrists’ motivation for this rejection was patient secrecy. Similar to the experience of this mother, all other mothers described how they have felt—and in reality were—excluded from psychiatric care. Psychiatrists and other professionals all referred to patient secrecy or had not responded to phone calls or other attempts at contact. This exclusion of the parents in their children’s medical treatment and the psychiatrists’ unwillingness to listen to the parents’ experiences was something that all mothers criticised, not least because they as parents have had unique insights into how the medication has affected their children. For example, one recurrent theme in the mothers’ stories was that they at once could tell the side effects of the medication, if a medication did not work or if their child did not follow the prescriptions. This knowledge was neither requested nor wanted by the psychiatrists, even though the few times the mothers had succeeded in contacting their children’s doctors, they had actually provided the psychiatrists with vital information:

The doctors didn’t talk to us either. No one contacted us or asked us about anything. Nevertheless, we phoned and asked for an appointment with the doctor (…) and I told this to the doctor [how the symptoms of the illness emerged] and then her or if it was somebody else said “This you’ve told us is good. This helps us to come up with the correct diagnoses”. Then I thought why couldn’t they phone then and ask us who live with him and have known him since he came to this world. Why? I think this is so bad. Why no cooperation?.

Consequently, when psychiatric care took responsibility for their children, the mothers were excluded from the treatment, from discussions about the purpose of the treatment and from plans for the future. At the same time, the mothers described how they were expected to take on the responsibility for their children when there were no spare beds or when the hospital staff wanted to minimise the number of patients during weekends and holidays.

Those mothers who had children who developed the illness when they were teenagers argued that the transfer from child and youth psychiatric care to adult psychiatry marked a clear change both in terms of the quality of the care and in their influence as parents. Formally, their responsibility ended when the child reached the age of 18, but in practice, their caring responsibilities continued. In this context, the mothers stated that there was minimal understanding from the side of the professionals of this situation. Instead of being asked for participation and formally including them in the care of the child, professionals within psychiatric care excluded the mothers with the argument that this was necessary for the son’s or daughter’s liberation from the parents and in order to become an adult.

Another experience the mother shared with us was that their knowledge of their children was seldom regarded as an asset in the care of the child. On the contrary, their knowledge was more often considered a part of the problem. One frequent theme in the mothers’ stories of encounters with psychiatric care was therefore about endless battles about not being marginalised as parents or even losing access to their children. The older mothers and one younger mother, whose daughter at one point was admitted to forensic care, also described how they sometimes have been forbidden to visit their children, or only allowed to visit them under surveillance.

At Beckomberga [one asylum that now has been closed] the head of the gang that had (…) distributed the illegal drugs often came to visit and then I told the chief physician that perhaps it wasn’t that suitable because he had provided the youths with drugs. After a while, I was not allowed to visit her. However, he (the head of the gang) was allowed. Then the chief physician said, “Well, she’s much happier when he has visited her. And when you’ve visited her she’s always a little bit worried”. Stupid doctor, really, that doesn’t speak to me and ask me about what I’m telling him.

## Encounters with Community Care

The mothers more often described encounters with social care and social workers as positive experiences. The mothers particularly appreciated the possibility for their children to have a contact person or a case manager who assisted their children with social activities. The possibility for their child to have his or her own flat in a group living context was also described as a positive experience, although this form of service had not always been without problems.

And when she arrived there at the group living, you noticed that they preferred to know when we parents would visit. Really, it’s not as living in your own apartment and you can visit your children whenever (…) you could feel that you weren’t really welcome at all occasions.

As social services were non-compulsory and directed towards support in daily living such as help with arranging an apartment, social activities and possibilities to work, the mothers experienced social workers as much easier to deal with than psychiatrists. However, there were also similarities when it came to personal interactions. In relation to psychiatric care, the mothers had experienced a professional discourse that excluded the parents’ knowledge of their children, but in social care services, the mothers faced another discourse of sanism that also excluded them. Recurrent in the mothers’ stories of encounters with the social services was that their knowledge of their children in terms of what they liked to do, how they liked to have things and what they were interested in, were rejected if this did not fit with the discourse of what ‘normal’ people do.

In short, the mothers described this discourse of ‘the normal’ as a set of norms and rules of how to furnish and clean an apartment, when and with whom you drink your coffee, how often it is suitable to see your parents and where you go on holiday. One mother described the encounter with the sanism discourse prevalent within the social services as a discourse that characterised everything that was connected to their family life, their interests and hobbies, their travels and holiday plans and their eating habits as ‘weird’. Another mother, who for many years had to take on the entire caring responsibility for her adult son when he refused to let social services enter his apartment, ironically said that if a person manage to do his laundry, then he is ‘normal’ according to the social services.

Our son, he refused to open the door so then they [social services] couldn’t enter. No. So in that way, he managed to escape all forms of care and just sat there (…). I had to take care of him by myself for several years. He had to come here, eat and take a shower and change clothes and all that, because they were not allowed to enter. Then they would teach him how to do the laundry, and that is the first and only criteria society puts up for the ill. If you can do your laundry, then it’s completed.

The mothers’ stories also often included searching for an option or an alternative to publicly organised care. Over the years, the mothers and their children had explored alternative forms of therapy, such as meditation, herbs and spiritual forms of therapy that had functioned as parallel supports to public care. The church and other spiritual societies were often mentioned as assets, not at least because the mothers described how it was nearly impossible for their children to access conversational therapy. Instead, their children’s routes through the public care system were characterised by never-ending changes in medication and continuous experiments with medicine dosages.

## Looking Ahead—Thinking About the Future

Our final theme in the interviews with the mothers was about reflections on the future. This theme came out somewhat differently depending on the age of the children. Most of the children were about to reach their 40 s or their 50 s, and the mothers mentioned that they used to worry more when their children were in their 20 s. During that age, their children’s deviations from the normal life course were so apparent, as their children for example never began to study at university or had employment like ‘normal’ children. In addition, in most cases their children never established their own families, something that was also considered ‘abnormal’. However, the mothers argued that as the years have gone by, the social pressure on their children has diminished, which has been a release both for them and for the mothers. Nevertheless, for the oldest mothers, new worries were occurring, as they were afraid of what would happen to their children when they could no longer be there for them. The worries were partly about daily living and the financial situations of their children, but above all, they feared that their children would be isolated and completely alone when they were gone.

Another theme in the mothers’ thoughts of the future was that their relationship with their child had changed in relation to ageing. Even though the mothers always had been there for their children, this had not meant that their relationship was frozen in the past. On the contrary, many mothers mentioned how the relationship had developed into a close friendship. For example, one mother described how she looked at her relationship with her daughter as a reciprocal friendship based on unique experiences. The mothers also argued that their children’s maturities had affected the relationships between them. The older the children have become, the more equal the relationship has become. Therefore, the negative experiences from the onset of the illness and the daily worries have disappeared into the background and been replaced with personal qualities, inspiring conversations and activities together. One mother put it this way: ‘In time you become the same age as your children’.

The historical period and context of the child’s illness are of course important. The oldest mothers encountered a hospitalised and psychiatric culture and environment that were both hostile and terrifying. They also mentioned that as the years have gone by, they have observed the development towards more friendly and community-based services, but they also concluded that ‘unfortunately, this came too late for our son/daughter’.

## Discussion

In this study, we have illustrated how discourses and practices of sanism have affected families and how mothers have fought against both oppression and discriminatory actions. As the social community and the context of psychiatric and social care are permeated with norms that favour sanity, stigma is still attached to mental illness. Prejudice and ignorance cause everyday discrimination of families with experiences of mental illness and psychiatric care.

Regardless of the age of the mothers we interviewed, they all described in different ways how the everyday discrimination of people with mental illness, which Poole et al. (2012) define as sanism, also has spilled over into their lives. This became evident when their children firstly were diagnosed with a mental illness. Because of negative attitudes and humiliating comments, the mothers questioned themselves as not being competent parents; they started to wonder what they had done wrong and their self-accusations were supported by people in their social surroundings. For example, the mothers had often experienced that they were labelled as incompetent mothers in an overall ‘weird’ family.

Even though the mothers we interviewed described how they in time had adapted to the problems of their children and thereby developed their own strategies to tackle sanism, the mothers were continually confronting new problems and new situations along the way. In Sweden, one of the most neglected issues of sanism is the economic consequences for all parties involved. As demonstrated by the life stories of the mothers we interviewed, people with mental illness in Sweden are usually categorised as unable to work and therefore most of their lives depend on social welfare or disability pensions. Due to the low pension levels, the mothers we interviewed had, alone or together with the father of the child, been forced to take on a lifelong responsibility to financially provide for the child, even though the child was an adult and in his or her 40 s or the 50 s at the time of the interview. In some cases, this responsibility even included grandchildren. The oldest mothers also described how this life-long financial responsibility threatened their own financial security in old age.

Another central theme in the mothers’ stories was the importance of a social network to be able to develop strategies to deal with sanism. Whilst a few have been able to include their experiences in previous networks, the most common strategy was to find new networks, primarily based on their children’s mental illnesses. Nearly all mothers emphasised that the user organisations have been vital to them, as, through the user organisations, they have been able to speak about their experiences and been met with empathy and understanding by persons in similar situations.

Concerning publicly organised mental health care services, the mother’s life stories indicate that things have changed for the better. Asylums and psychiatric wards are being replaced with community care and social services that offer support and help with living arrangements, jobs and social activities. However, though the mothers did not experience community care to be as harsh as psychiatric care, social services were sometimes experienced as too rigid. For example, the mothers argued that community care functioned by a principle of preference for the sane that in positive terms implied that people with mental illness should have equal opportunities. At the same time, however, the mothers had experiences of how professionals in social services sometimes applied ‘normal behaviour’ as a template for how their children should live their lives. Thus, to the mothers, this idea in community care of what normal people do and do not do came out as forced values based on what social service professionals thought constituted a sane and normal life. These experiences suggest that professionals in social services ought to consider how to include and accept differences within what is considered ‘normal’.

Overall, confrontations with ideas, values and perceptions of what constitutes a ‘normal’ life for their children were essential parts of the mothers’ life stories—confrontations that also affected how the mothers evaluated their own lives. In addition, the mothers frequently described how their children have been labelled as ‘abnormal’, ‘wrong’ or even ‘an accident’. This negative and degrading naming of their children also made the mothers feel excluded and sometimes even as outcasts in social interactions with friends, neighbours, colleagues and relatives. Negative attitudes and comments from surroundings and professionals have also forced the mothers to go through painful and prolonged processes of self-accusation for not having given enough love, care, support and help in different stages of their children’s lives.

Although the mothers’ stories confirmed the conclusion that most families adjust to the situation of having a mentally ill child (Schön et al. 2006; Topor 2006) and that family members reach new understandings of each other (Veltman et al. 2002), the mothers’ narratives also revealed important aspects of changed relationships over their life spans. As the mothers aged and when they themselves became in need of everyday support for daily life activities, it was the so-called disabled child that took the role as the family carer. Thus, in the mothers’ narratives, the relation between them and their children became reciprocal in old age.

Another insight from the interviews is alternative understandings of recovery from mental illness. Recovery is generally understood as returning to an ideal state of normal life, and in practice summarised as the ability to work and have employment. In contrast, studies by Reupert et al. (2015), Topor et al. (2006) and Schön et al. (2009) have highlighted intimacy and reciprocity among family members as important to the recovery process. In the mother’s narratives, this alternative understanding of recovery was also confirmed, in particular in the descriptions of how the mothers and children have grown old together.

Finally, we want to point out that our sample consisted of mothers engaged in the two main user organisations in the field of psychiatric care in Sweden. In this sense, they can be seen as privileged because they have had the time and possibility to give voice to their experiences. However, in this context, we would like to address the ongoing focus on user participation in mental health services. Despite the increasing focus on user participation in contemporary mental health services, professionals still generally take on a top-down role as teachers; i.e., to educate service users and their families on various issues related to SMI. However, the life stories of the mothers in this study suggest that a bottom-up perspective that includes the entire family network is equally important to improve the care and support of people living with SMI. Family members have important experiential knowledge about living with mental illness and psychiatric and social services. Consequently, if the user perspective is to be taken seriously, this knowledge must also be included to advance research and practice.
